# Retracted: The Effect of *Elephantopus scaber L.* on Liver Regeneration after Partial Hepatectomy

**DOI:** 10.1155/2017/4561243

**Published:** 2017-11-26

**Authors:** Evidence-Based Complementary and Alternative Medicine

Evidence-Based Complementary and Alternative Medicine has retracted the article titled “The Effect of *Elephantopus scaber L.* on Liver Regeneration after Partial Hepatectomy” [1]. The article was found to contain images with the following concerns: The tubulin bands in Figures 2(a) and 2(c) are the same; in Figure 3(a), in the top row there is a vertical line, the first row has a uniform background in the first six lanes, the second row is highly similar to the third row though with increased contrast and the height reduced, and the tubulin and HGF lanes do not align; in Figure 3(b), the top row has no background noise in the first three lanes, the background has irregularities in the fifth lane from the right, and the tubulin lanes do not align with the rest; in Figure 3(c), the fourth row has irregularities in the background in the first six lanes, and the tubulin lanes do not align with the rest; in Figure 5(a), the top row has a horizontal line across the band, there are two vertical lines in the tubulin row between the first and second lanes and between the fourth and fifth lanes. The authors provided corrected figures, which are available as Supplementary Materials, but could not provide the underlying blots.
